# New Materials and Their Applications: Perspectives in Restorative Dentistry and Endodontics

**DOI:** 10.3390/ma15175846

**Published:** 2022-08-24

**Authors:** Xi Wei, Sui Mai

**Affiliations:** Department of Conservative Dentistry & Endodontics, Guanghua School of Stomatology, Hospital of Stomatology, Sun Yat-Sen University, Guangzhou 510050, China

“New Materials and Their Application: Perspectives in Restorative Dentistry and Endodontics” is a new open Special Issue of Materials, which aims to publish original and review articles on novel scientific research that closely relates to new dental materials and their synthesis, function, characteristics, and applications. As new materials and perspectives on dental materials keep emerging, enabled by contemporary advanced science and technology, this realm is becoming increasingly broad.

The essence of restorative dentistry and endodontics is the preservation of natural teeth. New materials in restorative dentistry and endodontics are the forefront of contemporary dental materials, which focus mainly on the preservation of the pulp and the teeth. Their specific functions incorporate caries prevention and treatment, living pulp preservation, damaged pulp management, pulp regeneration, remineralization of demineralized dental tissue, and esthetic restoration of defected tooth to recover function and appearance. In this area, the materials range from synthetic polymers, metals, and bioceramics, which are used in restoration, pulp-capping, root canal filling, and repairing. Innovative technology and pioneering discoveries of materials in the field are needed that can help to tackle problems such as polymerization shrinkage, the duration of resin-based materials, and the biological toxicity of certain materials. Specifically, advancements are essential to modify materials and give them different properties, so as to improve their mechanical properties, antibacterial properties, mineralization properties, self-healing properties, and regeneration properties according to the demands of clinical application [1,2].

In the contemporary technology-driven world, with the development of artificial intelligence, 3D printing, and CAD/CAM (computer aided design/computer aided manufacturing) technology, dental materials are facing enormous new challenges. The advent of machinable materials has significantly catalyzed the promise of digital technology. However, esthetic appearance, wear resistance, and dimensional accuracy are the main clinical limitations restricting the application of the cutting-edge technique, which urgently calls for further studies [3].

The research interest of the section “New Materials and Their Application: Perspectives in Restorative Dentistry and Endodontics” includes, but is not limited to, original research articles and reviews that emphasize the latest advances and perspectives of dental materials in restorative dentistry and endodontics.

## References

[1] Fugolin A.P.P., Pfeifer C.S. (2017). New Resins for Dental Composites. J. Dent. Res..

[2] Primus C.M., Tay F.R., Niu L.N. (2019). Bioactive tri/dicalcium silicate cements for treatment of pulpal and periapical tissues. Acta Biomater..

[3] Della Bona A., Cantelli V., Britto V.T., Collares K.F., Stansbury J.W. (2021). 3D printing restorative materials using a stereolithographic technique: A systematic review. Dent. Mater..

